# Influence of iRoot SP and mineral trioxide aggregate on the activation and polarization of macrophages induced by lipopolysaccharide

**DOI:** 10.1186/s12903-018-0511-9

**Published:** 2018-04-02

**Authors:** Zhenglin Yuan, Xiaodan Zhu, Yuhong Li, Ping Yan, Han Jiang

**Affiliations:** 1 The State Key Laboratory Breeding Base of Basic Science of Stomatology (Hubei-MOST) and Key Laboratory of Oral Biomedicine Ministry of Education, School and Hospital of Stomatology, Wuhan University, 237 Luoyu Road, Wuhan, 430079 People’s Republic of China; 2 Department of Stomatology, Union Hospital, Tongji Medical College, Huazhong University of Science and Technology, Wuhan, China; 3 Department of Periodontology, School and Hospital of Stomatology, Wenzhou Medical University, Wenzhou, China

**Keywords:** iRoot SP, Mineral trioxide aggregate, Polarization, Macrophages, Lipopolysaccharide

## Abstract

**Background:**

Biomaterials could affect the inflammation reaction and wound healing via the activation and polarization of macrophages. However, the influence of iRoot SP and mineral trioxide aggregate (MTA) on macrophage polarization under inflammatory conditions was not reported although these two root filling materials have been applied extensively in patients undergoing endodontic treatment. Therefore, the present study aimed to explore the mechanism how iRoot SP and MTA affect the cell behavior of RAW 264.7 macrophages when stimulated by lipopolysaccharide (LPS) in vitro.

**Methods:**

The gene expression of three main related pro-inflammatory cytokines (IL-1β, TNF-α, IL-6) was examined by quantitative real-time polymerase chain reaction (qRT-PCR) in RAW 264.7 macrophages when stimulated by iRoot SP and MTA in the presence of LPS. The protein expression of the M1 and M2 phenotype specific markers, CD11c and CD206, was assessed by immunofluorescence and flow cytometry in RAW 264.7 macrophages.

**Results:**

LPS promoted the expression of IL-1β, TNF-α, and IL-6 in RAW 264.7 macrophages as compared to the control group. Both iRoot SP and MTA were significantly able to enhance the expression of IL-1β, TNF-α, and IL-6 in RAW 264.7 macrophages as compared to LPS group. LPS could increase the expression of CD11c as compared to the control group while iRoot SP and MTA were able to enhance the expression of both CD11c and CD206 as compared to LPS group.

**Conclusions:**

iRoot SP and MTA could potentially promote the release of pro-inflammatory cytokines in RAW 264.7 macrophages and induce into M1/M2 phenotype when cultured with LPS.

## Background

The primary virulence factor of the gram-negative anaerobic bacteria, lipopolysaccharide (LPS), is involved in the initiation and development of the apical periodontitis [1, 2]. It is known that macrophages play a crucial role in the host response to LPS which induces the activation and polarization of macrophages [3]. It also activates the synthesis and release of pro-inflammatory cytokines such as IL-1β, TNF-α, and IL-6 that trigger the innate immune reaction and promote the periapical bone resorption [4–6]. Given that the endodontic material directly interacts with the inflammatory periapical tissue during endodontic treatment, it has great significance to elucidate the underlying mechanism that affects the biological behavior of the LPS-activated macrophages. This will be greatly helpful in assessing the role of endodontic material in the pro-inflammation response and tissue regeneration in periapical disease.

Due to its superior characteristics, mineral trioxide aggregate (MTA) has been extensively applied in various dental therapies [7], especially in repair of root performation and root-end filling. It may potentially promote the tissue repair and inhibit the osteoclast differentiation through the activation and polarization of macrophages. It inhibited the osteoclast differentiation of RAW 264.7 macrophages [8] and the mouse bone marrow macrophages [9]. The osteoclast quantity changes and bone resorption caused by LPS was also decreased by MTA in vivo [9]. It was observed that pulp capping with MTA in rat could induce the accumulation of M2 phenotype macrophages under the degenerative layer and the initial phase of healing [10]. Furthermore, the implantation of MTA into the back of the rats could enhance the polarization of M2 phenotype macrophages and the would repair [11]. As a novel pre-mixed bioceramic, iRoot SP was introduced into endodontic application due to its superior biocompatibility [12, 13], excellent antimicrobial efficacy [14], strong sealing ability [15], and potential tissue repair capacity [16, 17]. Although the potential mechanism that iRoot SP possesses these biological properties is unclear, it has been confirmed that the biological properties of Endosequence BC sealer, a bioceramic endodontic sealer, was relevant with its physicochemical properties such as high pH, greater release of calcium ion, and hydroxyapatite formation [18, 19]. Given that MTA, iRoot SP and Endosequence BC sealer are calcium silicate-based bioceramic, so maybe the physicochemical properties of MTA and iRoot SP are also associated with their physicochemical properties. However, the mechanism how iRoot SP influence the activation and polarization of macrophages has not been reported. In order to elaborate the underlying mechanism, we analyzed the biological effects of these two bioceramic materials on macrophages.

So the present study aimed to investigate the mechanism how iRoot SP and MTA affect the activation and M1/M2 phenotype polarization in macrophages induced by LPS. The present study hypothesized that these two bioceramic materials could increase the expression of the pro-inflammatory cytokines in RAW 264.7 macrophages when induced by LPS, in the meanwhile, both of them are able to induce RAW 264.7 macrophages into M1/M2 phenotype when stimulated by LPS.

## Methods

### Sample preparation

iRoot SP (Innovative BioCeramix Inc., Vancouver, Canada) and MTA (Dentsply, Tulsa, OK) were prepared according to the protocol mentioned in our previous article [20]. The sample was placed into a sterilized mold (5 mm in diameter and 3 mm in thickness) for 24 h at 37 °C and 5% CO_2_ in a cell culture incubator. It was further incubated for 24 h in Dulbecco’s Modified Eagle’s Medium (DMEM; Invitrogen, Carlsbad, CA, USA). Subsequently, the medium was collected and filter sterilized through 0.22 μm pore size membrane. The filtered medium was termed as “iRoot SP extract” or “MTA extract”.

### Cell culture

RAW 264.7 macrophages (American Type Culture Collection, ATCC) were cultured in DMEM with 10% fetal bovine serum (FBS;Gibco, Grand Island, NY, USA) and antibiotics (100 U/mL penicillin G and 100 μg/mL streptomycin) at 37 °C with 5% CO_2_. The cells were classified into four groups: (1) control group: Cells cultured with the regular medium; (2) LPS group: Cells stimulated with 10 ng/mL of ultrapure LPS from *Escherichia coli* (InvitroGen, San Diego, CA, USA); (3) iRoot SP group: Cells stimulated by iRoot SP extract medium and 10 ng/mL LPS; (4) MTA group: RAW 264.7 macrophages cultured with MTA extract and 10 ng/mL LPS. Each group was cultured for 24 h prior to the following experiments.

### qRT-PCR

RNA was isolated from cells using TRIZOL Reagent (Invitrogen) and was reverse transcribed to cDNA using Oligo dT and ReverTra Ace (TOYOBO, Japan). Real time PCR was performed using *Power* SYBR™ Green PCR Master Mix (Applied Biosystems™) on Applied Biosystems 7500HT Sequence Detection System (Applied Biosystems, Foster City, Calif.). The primer sequences were listed in Table 1. The expression data was analyzed with the △△Ct method. The experiment was performed in three independent experiments and each in triplicate.

**Table 1:** The primer sequence of GAPDH, IL-1β, TNF-α, IL-6

Genes	Primer Sequence
GAPDH	5’-CATGTTCCAGTATGACTCCACTC-3′ (forward) 5′- GGCCTCACCCCATTTGATGT-3′ (reverse)
IL-1β	5’-GCAACTGTTCCTGAACTCAACT-3′ (forward) 5’-ATCTTTTGGGGTCCGTCAACT-3′ (reverse)
TNF-α	5’-CCTCCCTCTCATCAGTTCTA-3′ (forward) 5’-ACTTGGTGGTTTGCTACGAC-3′ (reverse)
IL-6	5’-GAGGATACCACTCCCAACAGACC-3′ (forward) 5’-AAGTGCATCATCGTTGTTCATACA-3′ (reverse)

### Immunofluorescence

Cells were cultured for 24 h after seeded on glass coverslips and then 2.5% bovine serum albumin was used as blocking reagent for 1 h. After that the cells were conjugated with PE anti-mouse CD11c antibody (Biolegend, San Diego, CA, USA) or PE anti-mouse CD206 antibody (Biolegend) at 25 °C for 1 h. The samples on the coverslips were gently mounted with 10 μL DAPI mounting medium (Thermo Fisher Scientific, Waltham, MA, USA). The coverslips were sealed with the nail oil and images captured with Leica TCS SP5 confocal microscope.

### Flow cytometry

The cell pellet was acquired after centrifugation and washed twice with PBS containing 0.5% BSA. After that the cells were resuspended in PBS containing 0.5% BSA. Subsequently, the cells were homogenously mixed with PE anti-mouse CD11c or PE anti-mouse CD206. The cells were placed at 4 °C for 30 min and centrifuged. The cell pellet was washed twice and diluted in cold PBS to make it to be homogeneous. The collected homogeneous sample was analyzed with a flow cytometer (Epics Elite ESP; Coulter, Tokyo, Japan).

### Statistical analysis

The data were represented as mean ± standard deviation. Statistical significance analysis was performed by one-way analysis of variance followed by a Tukey’s test. *P* < 0.05 was considered as significant.

## Results

### Gene expression analysis

**Fig. 1:**
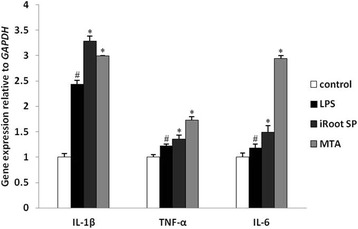
The mRNA expression of IL-1β, TNF-α, and IL-6 when RAW 264.7 macrophages were cultured with LPS, iRoot SP or MTA. GAPDH gene was chosen as reference gene. # refers the significant difference compared to control group (*p *< 0.05), * represents the significant difference compared to LPS group (*p *< 0.05)

In order to determine the mechanism how iRoot SP and MTA affect the activation of RAW 264.7 macrophages, we firstly explored mRNA expression changes of the pro-inflammatroy cytokines such as IL-1β, TNF-α, and IL-6 using qRT-PCR. Compared to the control group, the gene expression of IL-1β, TNF-α, and IL-6 was significantly increased when RAW 264.7 cells were treated with LPS (*P* < 0.05). Moreover, as compared to the LPS group, the expression of IL-1β, TNF-α, and IL-6 was significantly increased when cells were stimulated by iRoot SP or MTA medium (*P* < 0.05) (Fig. 1). However, it seems that iRoot SP enhanced the gene expression of IL-1β more than MTA while MTA increased the gene expression of both TNF-α and IL-6 more than iRoot SP. The result mentioned above displayed that both of these two bioceramic materials were able to promote mRNA expression of the pro-inflammatroy cytokines such as IL-1β, TNF-α, and IL-6.

### Protein expression analysis

**Fig. 2:**
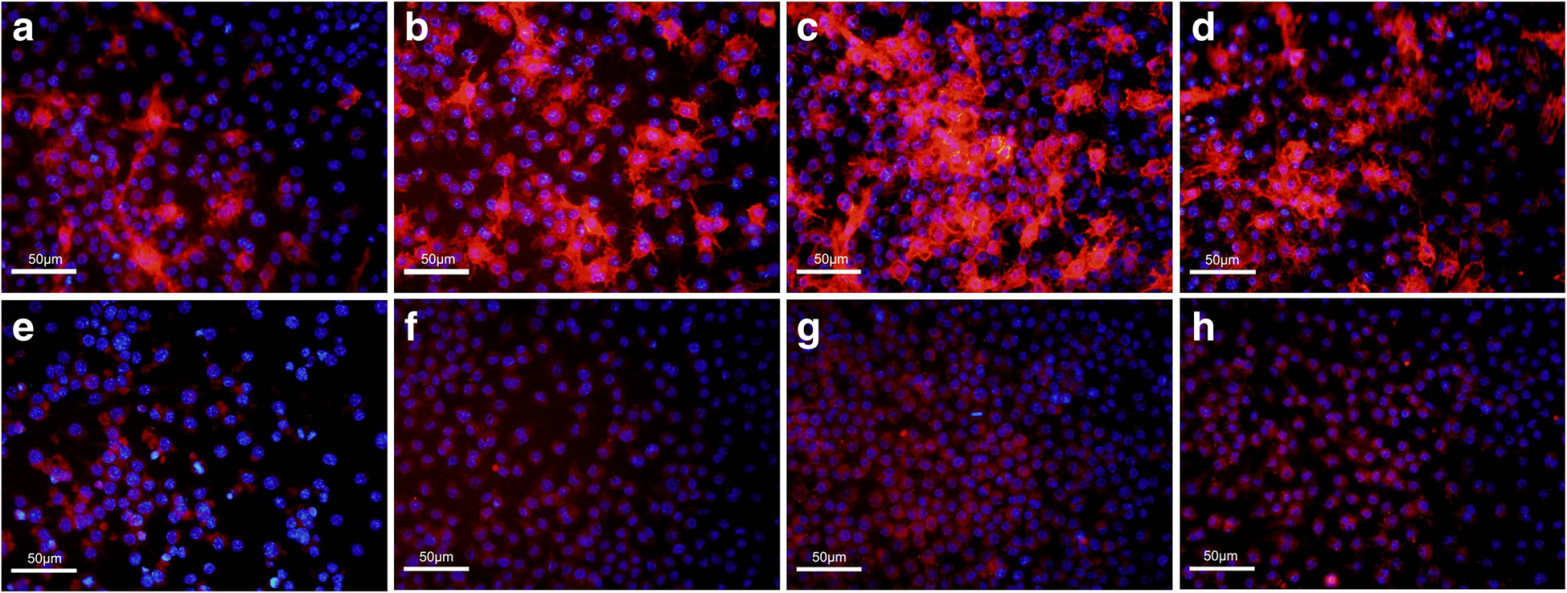
The protein expression of M1 phenotype specific marker CD11c and M2 phenotype specific marker CD206 with immunofluorescence. The cells were classified into four groups: (1) control group (**a**, **e**); (2) LPS group (**b**, **f**); (3) iRoot SP group (**c**, **g**); (4) MTA group (**d**, **h**). The blue color means the cell nuclear with DAPI staining, the red color means CD11c protein (**a**, **b**, **c**, **d**) or CD206 protein (**e**, **f**, **g**, **h**)

**Fig. 3:**
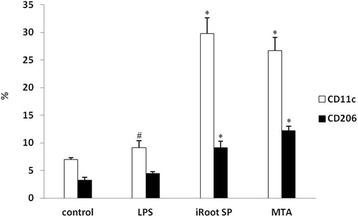
The percentage of CD11c/CD206 positive macrophages by flow cytometry. # means the statistic difference compared to control group (*p* < 0.05), * means the statistic difference compared to LPS group (*p* < 0.05)

To confirm how iRoot SP and MTA influence M1/M2 phenotype polarization of the cells, the protein expression of M1 phenotype specific marker CD11c and M2 phenotype specific marker CD206 was tested via immunofluorescence and flow cytometry. It was shown that, compared to control and LPS group, the fluorescence intensity of both M1 and M2 phenotype specific markers was substantial when cells were stimulated by iRoot SP or MTA medium (Fig. 2). The expression differences of M1 and M2 phenotype specific markers in all groups were quantified by flow cytometric analysis. And it indicated that LPS significantly stimulated the expression of CD11c (*P* < 0.05) of RAW 264.7 macrophages but not of CD206 (*P* > 0.05). On the other hand, the expression of both these two markers was remarkably enhanced in iRoot SP or MTA group as compared to LPS group (*P* < 0.05) (Fig. 3). The above results demonstrated that both of these two bioceramic material were able to induce macrophages into M1/M2 phenotype.

## Discussion

Macrophage activation and polarization were essential for the occurrence of the inflammatory diseases [21]. Two types of macrophages, M1 and M2, have been identified according to their phenotype and cytokines. M1 phenotype macrophages were principally responsible for the initiation of the immune response and the production of the pro-inflammatory cytokines while M2 phenotype macrophages modulated the inflammatory response and wound healing [22]. iRoot SP and MTA were widely used for the treatment of endodontic therapy and the interaction between the endodontic materials and macrophages will happen immediately. To investigate the biocompatibility of these two bioceramic materials with macrophages, our previous study found that iRoot SP and MTA were biocompatible with macrophages could affect macrophage M1/M2 phenotype in vitro [20]. As we know, the macrophage polarization was involved in the pathogenesis process in the apical periodontitis [23] and low doses of LPS could lead to persistent, non-resolving inflammation by the induction of persistent mild M1 phenotype macrophage mediated pro-inflammation and M2 phenotype macrophage mediated pro-inflammation mechanisms [24]. Moreover, bioceramic-based endodontic material such as MTA was effective in the treatment of severe apical periodontitis compared to gutta-percha [25]. Therefore, to investigate the possible mechanism how bioceramic-based endodontic materials promote the inflammation resolution and wound repair in periapical periodontitis, LPS-stimulated RAW 264.7 macrophages was chosen to mimic the inflammatory condition of periapical periododontics in vitro and to investigate the influence the iRoot SP and MTA on macrophage activation and M1/M2 phenotype polarization.

Firstly, we confirmed that the expression of IL-1β, TNF-α, and IL-6 was induced in RAW 264.7 macrophages when stimulated with LPS. The underlying molecular mechanism suggested that LPS could activate the macrophages mainly via Toll-like receptor 4 (TLR4) and subsequently promote the release of the pro-inflammatory cytokines such as IL-1β, TNF-α, IL-6, IL-8, and CXCL8 [1]. Surprisingly, both of these two bioceramic mateirals were able to significantly increase but not decrease the expression of the pro-inflammatory cytokines IL-1β, TNF-α, and IL-6. Therefore, iRoot SP and MTA could increase the expression of the pro-inflammatory cytokines in RAW 264.7 macrophages induced by LPS. These results seemed to contradict with the clinical application of iRoot SP and MTA in the repair of periapical lesions [26]. This may be attributable to the reinforcement of macrophage infiltration in the initial stage, which is gradually reduced after subcutaneous implantation of MTA in rat [27]. Similarly, it was revealed that MTA was able to stimulate the expression of IL-1β and TNF-α in the early stage, which declined eventually after subcutaneous implantation of MTA in mice, and the acute inflammatory response provided the suitable environment for the integration of the biomaterial [28]. As far as we know, this study first reported that similar to MTA, iRoot SP was capable of promoting the production of the pro-inflammatory cytokines and the initiation of acute inflammation response for the integration of endodontic materials and the subsequent influence on the wound healing of the periapical lesion.

The mechanism of iRoot SP potential to enhance the release of the pro-inflammatory cytokines is yet an enigma. However, it has been proven that the biological activity of bioceramic endodontic material was associated with its physicochemical properties, such as high pH, greater release of calcium ion, and hydroxyapatite formation [18, 19]. Because both iRoot SP and MTA are bioceramic endodontic materials, their similar effect on the release of the pro-inflammatory cytokines might be caused by their similar physiochemical properties. The calcium silicate could release both Ca and Si ions; the concentration of Ca ions decreased over a period while that of Si ions increased [29, 30]. The Si but not the Ca ions released from MTA/calcium silicate could provide the environment to enhance the expression of IL-1β in dental pulp cells when cultured with MTA [29]. This study only described the phenotype how iRoot SP and MTA affect the release of pro-inflammatory cytokines in RAW 264.7 macrophages but not investigate the underlying mechanism. Therefore, to clarify the relationship between these two materials and the pro-inflammatory cytokines’ secretion in RAW 264.7 macrophages, further experiments are necessitated to evaluate their physicochemical properties.

To further clarify the mechanism how iRoot SP and MTA influence macrophage activation and M1/M2 phenotype, the protein expression of CD11c (the unique marker of M1 phenotype macrophage) and CD206 (the unique marker of M2 phenotype macrophage) was detected via immunofluorescence and flow cytometry. The immunofluorescence results suggested that both CD11c and CD206 were localized on the cell membrane of the polarized RAW 264.7 macrophages. CD11c and CD206 expression was quantified by flow cytometry and the results demonstrated that LPS stimulation could primarily promote M1 phenotype polarization, which was identical to the previous study [31]. Interestingly, both of them could were induce into M1 and M2 phenotypes polarization. Therefore, our study revealed that iRoot SP and MTA are able to promote M1/M2 phenotype polarization in macrophages when cultured with LPS. As we know, M1 macrophages were essentially involved in the initiation of the pro-inflammatory response and the elimination of the pathogens while M2 macrophages were responsible for the regulation of the inflammatory response and wound healing. The implantation of MTA into the back of the rats could induce the accumulation of M2 macrophages [11] that participated in the initial healing process after pulp capping in rat with MTA [10]. Our results found that both of these two bioceramic materials could induce macrophages into M2 phenotype polarization, which was consistent with the above-mentioned previous studies. Moreover, the present study also discovered that both of them could promote M1 phenotype macrophage polarization, which in turn could enhance the production of pro-inflammatory cytokines, as observed qRT-PCR.

The imbalance of M1/M2 polarization was associated with various inflammation conditions [32]. LPS, the main virulence factor for apical periodontitis, could promote the M1 phenotype polarization and their predominance would lead to persistent inflammation [24]. It can also explain the clinical phenomenon that the LPS level is associated with the severity of the periapical lesions [2]. We observed that iRoot SP and MTA had the capacity to promote M1 and M2 phenotype in macrophages. The M1 phenotype induced by these two materials implies the enhancement of the acute inflammatory response. A normal inflammatory response usually comprises of the initial acute response followed by the anti-inflammatory response for wound healing and tissue repair [24]. Therefore, it suggested that both of these two bioceramic materials potentially transformed chronic inflammation condition into an acute response. This might be a plausible explanation for MTA as an effective endodontic material for the treatment of the persistent apical periodontitis [26]. Therefore, both of them could rectify the imbalance of M1/M2 phenotype polarization in RAW 264.7 macrophages induced by LPS, which systematically rationalized their efficient usage in endodontic treatment as the ideal endodontic materials.

In summary, iRoot SP and MTA were able to promote the release of the pro-inflammatory cytokines of RAW 264.7 macrophages in the presence of LPS stimulation. Moreover, both of them could promote M1/M2 phenotype macrophage polarization and modulate the imbalance of M1/M2 phenotype polarization induced by LPS. Therefore, the current study provides the insight into the mechanism of bioceramic-based endodontic materials affecting the pathogenesis of the apical periodontitis.

## Conclusion

MTA and iRoot SP were able to effectively enhance the gene expression of several pro-inflammatory cytokines IL-1β, TNF-α, and IL-6 in RAW 264.7 macrophages when cultured with LPS. Moreover, both of these two endodontic materials had the capacity to induce M1 and M2 phenotype polarization in RAW 264.7 macrophages induced by LPS.

## Abbreviations

DMEM: Dulbecco modified eagle minimum essential medium
FBS: Fetal bovine serum
LPS: Lipopolysaccharide
MTA: Mineral trioxide aggregate
qRT-PCR: Quantitative real-time polymerase chain reaction
TLR4: Toll-like receptor 4
